# Notes and News

**Published:** 1895-07-27

**Authors:** 


### Notes and News.

(Collected and Communicated.)

In the United States additions are to be made to the Buffalo General Hospital and to the New Jersey State Hospital, and a new small-pox hospital is to be erected for the State of Columbia.

The late Rufus Waterhouse has left 200,000 dollars to St. Luke's Hospital, New York, for the maintenance of a ward for consumptive seamstresses or those dependent upon them. It is to be named the "Mary S. Waterhouse Memorial Ward."

A school of medical hydrology has been opened at Luchon in France. It possesses a good laboratory, and nine professors on the teaching staff. The instruction is free to meritorious students of the various faculties and certain other persons. The teaching is directed by Dr. Garrigon, of Toulouse.

A memorial bed to Dr. William Cholmeley, late Consulting Physician, has been presented to the Great Northern Central Hospital by the ladies' association connected with the institution. The collection for the purpose amounts to £1,050, and shows a substantial and fitting appreciation of Dr. Cholmeley's labours.

The fact, as proved by the cases of small-pox originating at Blackfriars, that Salvation Army shelters can be dangerous to the health of the community, is very disquieting. The majority of the thirteen cases occurring in the vicinity of this shelter are directly traceable to it. The medical officer of health could only obtain admission to inspect the shelter when armed with a warrant and the threat of a large police force to enforce this authority. He found great over-crowding and absence of proper ventilation. The vestry of St. George's will serve a notice to the occupier of the shelter to abate the nuisance, and we trust that Dr. Waldo will continue his effective vigilance, especially during the winter months, when the demands for admission must be greater than now.

A new hospital is about to be erected at Aberdeen for the benefit of the sick poor in that place and the surrounding parishes. The late Mr. James Fleming made a bequest of £6,000 for the purpose of purchasing a site, erecting the hospital, and providing partially for its maintenance. The institution is to be called the James Fleming Cottage Hospital. Paying patients are to be received when space permits, and paupers also under the same conditions, at the expense of the local authorities.

The programme of the meeting of the British Medical Association in London is now complete. The address on Medicine will be delivered by Sir William Broadbent; the address on Surgery by Mr. Jonathan Hutchinson; and that on Physiology by Professor Edward Schäfer. The scientific business of the meeting will be conducted in fifteen sections. Numerous entertainments and excursions have been arranged, and the Ladies' Reception Committee have organised special excursions for lady guests.

We recently received a complaint from a patient who had attended the Dental Hospital of London, that the hospital, supported by voluntary contributions, did not supply dental appliances free. As is our custom, we applied to the hospital authorities for an explanation, and we learn that at the Dental Hospital the appliance department is distinct, and carried on apart from the work of the hospital proper, and those requiring dentures are expected to secure help through friends to defray the expense, the Hospital Sunday Fund frequently affording useful help in this direction.

The friends and pupils of the late Dr. Alphonse Guérin propose erecting a monument to his memory in his native town of Ploërmel.—A bust of the late Dr. Dujardin-Beaumetz has been placed in the Cochin Hospital, Paris.—At Locerain a statue has been erected to the memory of Father Damien, and at Paris a statue has been erected to Boussingault, in memory of his services in securing improved sanitation for the city.—Madame Jacolet, of Paris, has left 24,000 francs to found a bed in the Hospital Hotel Dieu de Point-a-Pitre in the Guadaloupe; and Monsieur Théodore Bourge, of Salerne, France, has left 200,000 francs for the erection of a hospital at that place.

The work of the hospitals and the large expenditure they entail is becoming generally understood, but the equally important work of convalescent aid is not so well realised. Above all, a visit to a convalescent home, help towards necessary surgical appliances, and clothing are required for patients leaving our London hospitals. Most hospitals now have their convalescent funds, but these are seldom found adequate. Taking St. Thomas's Hospital as an example, we see that upwards of £1,324 was expended on the most necessitous cases. This was more than the income by £200, and even with this reduction of capital it could not be said that every case that would have benefited by a change to country air after illness was able to secure it. St. Thomas's Samaritan Society may be regarded as one of the most affluent, yet even St. Thomas's could usefully spend much larger sums.

